# Developing and Testing a Framework for Learning Online Collaborative Creativity in Medical Education: Cross-Sectional Study

**DOI:** 10.2196/50912

**Published:** 2025-06-05

**Authors:** Shairah Radzi, Joo Seng Tan, Preman Rajalingam, Jennifer Cleland, Sreenivasulu Reddy Mogali

**Affiliations:** 1 Lee Kong Chian School of Medicine, Nanyang Technological University Singapore, 11 Mandalay road, Singapore, 308232, Singapore, 65 65923114; 2 Nanyang Business School, Nanyang Technological University Singapore, Singapore, Singapore; 3 Institute of Learning, Mohammed Bin Rashid University of Medicine and Health Sciences, Dubai, United Arab Emirates

**Keywords:** collaborative creativity, design thinking, medical education, social cultural learning, collaborative learning

## Abstract

**Background:**

Collaborative creativity (CC) is a social process of generating creative and innovative solutions to real-world problems through collective effort and interaction. By engaging in this process, medical students can develop abilities and mindset for creative thinking, teamwork, interdisciplinary learning, complex problem-solving, and enhanced patient care. However, medical students have demonstrated limited creativity, constrained by existing pedagogical approaches that predominantly emphasize knowledge outcomes. The increasing complexity of health care challenges necessitates a pedagogical framework for medical students to foster CC in a rapidly evolving professional environment.

**Objective:**

This study aimed to develop, test, and evaluate a new Framework for Learning Online Collaborative Creativity (FLOCC).

**Methods:**

FLOCC builds on established pedagogical approaches such as design thinking and integrates sociocultural learning methods (team-based learning [TBL] and problem-based learning [PBL]). It includes 4 individual asynchronous activities (empathy map, frame your challenge, turning insights into how might we questions, and individual brainstorming) and 5 collaborative synchronous activities (bundle ideas, list constraints, final idea, prototyping, and blind testing). In this cross-sectional study, 85 undergraduate medical students participated in 2 separate studies (study 1, n=44; study 2, n=41) involving health care and engineering sustainability problems. Learner acceptability was measured using a 31-item survey (using 7-point Likert scale) consisting of 4 factors (distributed creativity, synergistic social collaboration, time regulation and achievement, and self and emotions) and 3 free text questions. Free-text comments were subjected to the inductive thematic analysis.

**Results:**

Most students were positive about FLOCC, with distributed creativity and synergistic social collaboration factors receiving the highest mean percentages of “’Agree” (78/85, 92% and 75/85, 88%, respectively). These were followed by time regulation and achievement factor (68/85, 80%) and the self and emotions factor (59/85, 70%). Only time regulation and achievement was statistically significant (*P*=.001) between means of studies 1 and 2. Thematic analysis revealed 4 themes such as learning experiences, collaborative responsibilities, perceived skill development, and technical challenges.

**Conclusions:**

With effective time management, FLOCC shows potential as a framework for nurturing CC in medical students. Medical schools could provide the opportunity and environment that supports creative thinking; therefore, creativity-focused approaches could be integrated into the curriculum to encourage a culture of creativity for breakthrough solutions by future doctors.

## Introduction

### Background

Many jobs require graduates to engage in collaboration with other professionals, exhibit creativity in solving difficult problems and manage uncertainties in their careers. In medicine, doctors must work in teams to effectively manage patient care. They are also increasingly expected to work together to solve real-life health care challenges such as managing complex patient problems in a milieu where specialized medical services and manpower are scarce [1].

Over a century has passed since Flexner’s influential report [2], and medical education has undeniably experienced substantial changes, however, traditional medical education fail to cultivate the necessary creativity to handle difficult, ill-defined, or “impossible-to-solve” issues or problems which require multiple stakeholders to collaborate and innovate [3]. By default, pedagogical approaches in medical education tend to emphasize knowledge acquisition, retention, and problem-solving within a limited context [4]. In general, learning in medical school includes 2 types of knowledge: factual and procedural knowledge. Mann comprehensive analysis [5] underscores that sociocultural learning theories, rooted in the work of Vygotsky [6], present a valuable theoretical framework for shaping future medical education.

Significant ramifications arise from major modifications in medical education such as changes in our ways of knowing, changes in the discourse of medical education, changes in our view of medical education, which ultimately impacts the practice of medicine. The recent study by Fernández-Rodríguez found that the traditional teaching and assessment are still overused to the detriment of other teaching methods in medical education [7]. These methods typically measure the knowledge via written examinations focusing individual cognitive knowledge [8], not collaboration and creativity capabilities.

According to Guilford and Christensen [9], creativity is the ability and skill to create novel and innovative things. In Stein [10] classical paper, standard definition of creativity referred as “creative work is a novel work that is accepted as tenable or useful or satisfying by a group in some point in time” [10]. There is no single, agreed-upon definition of creativity due to its multidimensional nature [11]. But most agree that it requires the following 4 skills: the capacity for idea production (or “fluency”), attention to detail (or “elaboration”), originality (or “freshness”), and versatility (or “flexibility”) [12-14]. Kampylis and Valtanen [15] conducted an analysis of the 42 explicit definitions of creativity. Walia conceptualizes creativity as an ongoing act, irrespective of whether it results in a tangible creation [16].

In the context of education, collaborative learning is broadly defined as students working in groups of two or more sharing responsibility for tasks and products [17]. The fundamental factors for collaborative learning include respectful behavior, constructive feedback, a shared objective, acceptance of roles, engagement, and self-awareness [18]. The collaboration has been shown to foster the critical thinking by allowing students to debate ideas, engaging higher-order cognitive reasoning [19]. This highlights the importance of incorporating creativity and collaboration in medical curricula to address the problems in rapidly evolving health care system [20]. It also teaches students to view mistakes as opportunities for improvement when they are pushed to be creative [20].

### Collaborative Creativity

The reference [21] describe collaborative creativity (CC) as a social process that promotes the creative process in the form of relationships in completing group tasks [21]. The research on CC in medical education research is very limited, even though medical practices require teamwork and creative problem solving [22]. The recent systematic review differentiates CC (explicitly generating new ideas) from creative collaboration (focusing on how people work together, with creativity embedded) [12]. Hill et al [23] emphasize that innovation is not about solo genius [23] but is about the collective genius of collaborative problem solving, requiring 3 capabilities: creative abrasion (idea generation via debate), creative agility (rapid experimentation), and creative resolution (integrative decision-making) [24].

Several other factors impact CC, such as diversity of the team [25,26]; team interaction behaviors and conflict resolution [25,26]; task independence [27,28]; and the online or physical work space [29]. Online learning increased during the COVID-19 pandemic, demonstrating how digital platforms offer real-time cocreation and interaction. The information and communication technologies facilitates collaborative content creation through social contact among users and encourages user-to-user communication and cocreation [24]. Gundogdu and Merc [30] review found that web-based and cloud technologies, simulations, and smart tools help support technology-mediated CC. It was found that student performance is comparable to in-person learning [31]. In terms of questioning behavior and project performance, the online group performed better than the face-to-face group on collaborative tasks [32]. In the creative fields such as design and art, frameworks using technology to promote CC have become more common [33-35]. As higher education continues to expand online, medical programs must adapt to remain globally competitive and foster 21st-century competencies, including collective problem-solving, communication, and creativity [34].

While notable progress has been made in medical technology, education still lacks sufficient emphasis on creativity, which is crucial for problem-solving in the medical field [22]. Medical education often uses instructional methods such as traditional lecture-based, problem-based learning (PBL) and team-based learning (TBL). While the evidence indicates that structure of the PBL and TBL linked to learning enhancement, evidence of systematic attempts to foster CC remains limited [36-39]. Some researchers explored other creative methods in medical education such as brainstorming [40], concept maps [41], and storytelling [42] but CC still receives minimal attention. This is a critical gap in the literature, since CC (or lack thereof) can impact the medical professional-patient relationship [43], job engagement, satisfaction, and performance [44], and patient safety [45]. Thus, a need exists to create strategies and frameworks that incorporate the CC into medical education especially by leveraging online learning technologies to equip future medical professionals for the multifaced realities of evolving health care.

Therefore, the aim was to develop and test a novel design-based thinking framework for online collaborative creativity (FLOCC). Our research questions were: (1) What existing pedagogical principles could be utilized to create a novel FLOCC for fostering CC skills in medical students? (2) How could this FLOCC be operationalized? (3) What are students’ perceptions and attitudes toward the FLOCC?

## Methods

### Overview

**Figure 1.**
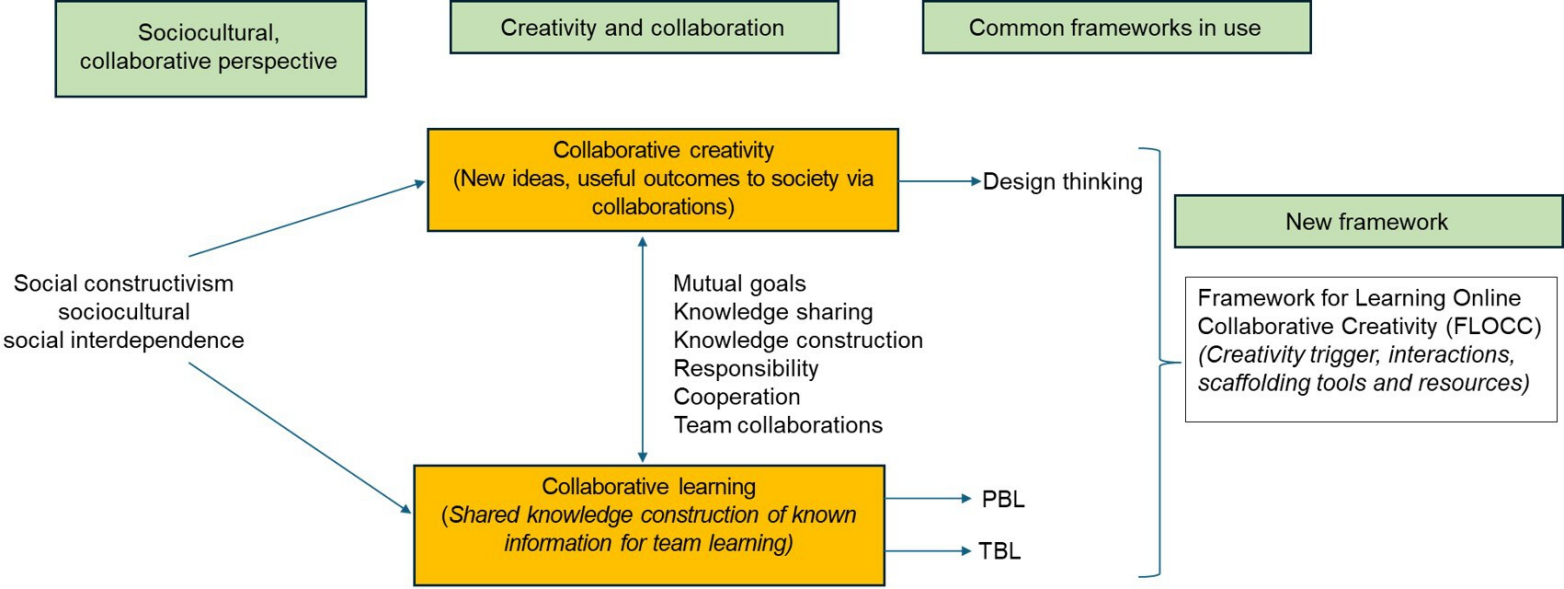
Conceptual underpinnings of the FLOCC: Framework for Learning Online Collaborative Creativity; PBL: problem-based learning; TBL: team-based learning.

This study reports the development and operationalization of a new FLOCC and its preliminary evaluation. The first stage in this process was to consider the conceptual underpinnings and structure of the FLOCC explicitly. The concepts underpinning FLOCC are depicted in Figure 1 and outlined below.

### Conceptual Underpinnings of FLOCC

FLOCC integrates the principles of rapid design thinking (RDT) with a sociocultural learning approach, encompassing elements of TBL and PBL. In both TBL and PBL learning takes place through interaction, negotiation, and collaboration in solving clinical problems. Hence, our goal is to identify the convergences of these methodologies within the framework of a collaborative, sociocultural learning grounded in Vygotsky’s sociocultural paradigm [6]. Dolmans et al [46] highlights how combining TBL and PBL could synergistically enhance problem solving skills, RDT framework further orients toward innovation and iterative solution creation. The current evidence supports the efficacy of TBL in enhancing the academic performance among the lower-performing medical students [47], while it may be greater demanding [36,38]. Conversely, PBL, has been linked to the development of a broader range of skills such as interpersonal skills, self-directed learning, planning and analytical skills [39]. However, some researchers have shown no difference between PBL and traditional learning in medical education [48] and caution that PBL effectiveness depends on frequent feedback [49].

Design thinking, defined as “a future-oriented method, process, toolbox or mindset” to promote collaborative and creative learning, adopts a human-centered problem-solving approach [50]. While design thinking has seen growing presence in Science, Technology, Engineering, and Mathematics education [51] but its application in medical education remains very limited. A recent review identified only 7 pertinent studies, 3 involving medical students [50,52]. However, while limited in number, these studies and recent opinion pieces suggest design thinking may be an appropriate framework to foster student, patient, and practitioner outcomes such as self-efficacy, learning experiences, and academic development [53,54]. Furthermore, using design thinking opens up a new area for investigation to help educators understand, measure, and assess the experiences of students to promote creativity [55], to create a new product and/or to establish a way of thinking about problems via the development of a novel product [50].

Common characteristics shared by sociocultural learning methods and RDT include goal orientation, team activity, brainstorming, and problem-solving. From these perspectives, learning is impacted at individual and collaborative levels through active engagement and shared goals [56]; building on knowledge through social interaction, constructive arguments and cooperation with others rather than passive learning [57]. From the social constructivist perspective, learning is more efficient in a group setting as knowledge develops as a result of social interaction and language use [58]; hence, students demonstrate more motivation and self-confidence in collaborative learning environments [59]. Through team conversations, students become more adept at identifying problems and pursuing solutions, leading to the process of problem-solving [60]. When individuals are accountable for building their own knowledge and communicating successfully with others, these activities can favorably influence CC [61].

In alignment with the sociocultural learning paradigm and echoing the integrative perspective of Dolmans et al [46], we propose combining TBL, PBL and RDT within FLOCC , harnessing the strengths of each method: TBL’s accountability and readiness assurance process [62], PBL’s “ill-structured” problem as a stimulus of thinking [63], and RDT’s creative process and quick prototyping [64].

Thus, FLOCC can be viewed as a hybrid pedagogical framework that combines key perspectives from PBL, TBL, and design thinking to tackle complex medical problems with CC skills.

### Structure of FLOCC

#### Overview

Process-based frameworks like FLOCC should capture the creative process and promote “mindful idea generation” in a sequential manner [65]. For this, 4 of the investigators (SR, TJS, PR and SRM), all experienced educators in TBL and PBL, adapted the 3 phases of FLOCC (inspiration, ideation, and implementation) from the RDT model, an accelerated version of the Stanford’s design thinking model, known as a “people-oriented problem-solving method” [66]. Through iterative discussions and consensus, RDT was chosen as it was especially useful in rapidly changing environments like Covid-19 whilst offering learners similar features to design thinking [64]. FLOCC includes 4 individual asynchronous activities (empathy map, frame your challenge, turning insights into how might we questions, and individual brainstorming) and 5 collaborative synchronous activities (bundle ideas, list constraints, final idea, prototyping and blind testing). These activities are incorporated into the 3 distinct phases of FLOCC, as explained in detail below:

#### Inspiration

Inspiration refers to defining a problem that can inspire opportunities for creative solution [64]. This phase requires understanding, observing, or listening to people with unmet needs. This means students would requires both communication skills and some domain knowledge [67] to empathize with end users’ unmet needs and be open to multiple possibilities. Problems here serve as the creativity trigger [68], akin to PBL, and students may benefit from preparatory materials to support the thought process, similar to pre-TBL preparation. Inspiration uses 2 individual learning scaffolds such as empathy map (I1) and frame your challenge (I2) [69]. These activities help individual learners systematically write down their insights, analyze stakeholders, contexts and think about potential solutions, forming a basis for ideas generation.

#### Ideation

The ideation phase reinforces observations and experiences recorded during inspiration via the generation of ideas and identification of potential solutions [67,70]. This phase is a dual-purpose scaffold to support individual learners and teams because it requires generating novel ideas or solutions about a topic at an individual level first, then team level. Here, students work individually using the “how might we question” (I3–turning their insights into opportunities for design via divergent thinking) and “Individual Brainstorming” (I4-to write as many ideas as possible based on I3-turning insights into opportunities for design via divergent thinking). Then students do team brainstorming where they combine and filter individual ideas through the team processes such as “bundle ideas” (T1) “list constraints” (T2) and final idea, (T3) and culminating in “prototyping” (T4). Rapid prototyping—a hallmark of design thinking encourages teams to translate their insights into tangible artifacts or solutions [71].

#### Implementation

Implementation refers to the process where designers test a solution by converting it into a working product and testing its feasibility and market value. This requires problem-solving which can happen via collaboration and mutual feedback [72]. Based on this, the last team-learning scaffold that is, “blind testing” (T5) created where teams assess the usability and feasibility of each other’s prototypes and provide feedback to refine them iteratively.

**Figure 2.**
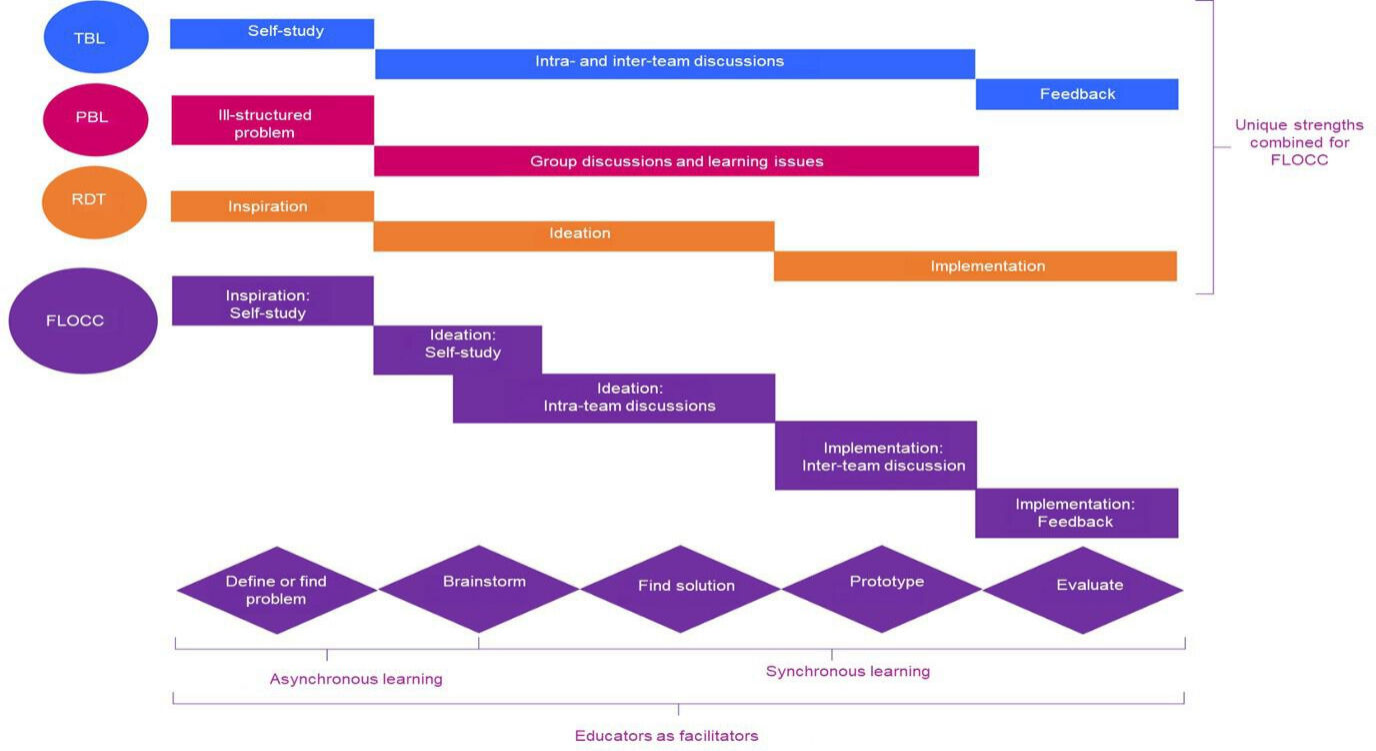
Hybrid characteristics of the FLOCC based on the strengths of TBL and PBL, and RDT. FLOCC: Framework for Learning Online Collaborative Creativity; PBL: problem-based learning; RDT: rapid design thinking, TBL: team-based learning.

Figure 2 depicts the stages of FLOCC and how it maps onto the characteristics of PBL, TBL and RDT.

### Operationalizing FLOCC

Following conceptualization, our next step was to operationalize FLOCC. The entire study was conducted online due to the COVID-19 pandemic. For operationalizing self-study in individual scaffolds, we provided a project website (Google Sites, Enterprise Standard, Google LLC) hosting explanatory videos about FLOCC and a downloadable Excel spreadsheet for collecting responses (Multimedia Appendix 1) of I1 to I4 individual scaffolds. The individual scaffolds were assigned in an asynchronous online learning format. An overview of the project website and a sample of individual Excel spreadsheets can be found in MultimediaAppendices 1  2, respectively.

For team scaffolds (T1 to T4), Google Sheets (Enterprise Standard, Google LLC, USA) was used to collect team responses simultaneously and in real-time. A sample of the Team Google Sheets can be found in Multimedia Appendix 3. In addition, a YouTube (Google) video on prototyping was also included [73].

**Figure 3.**
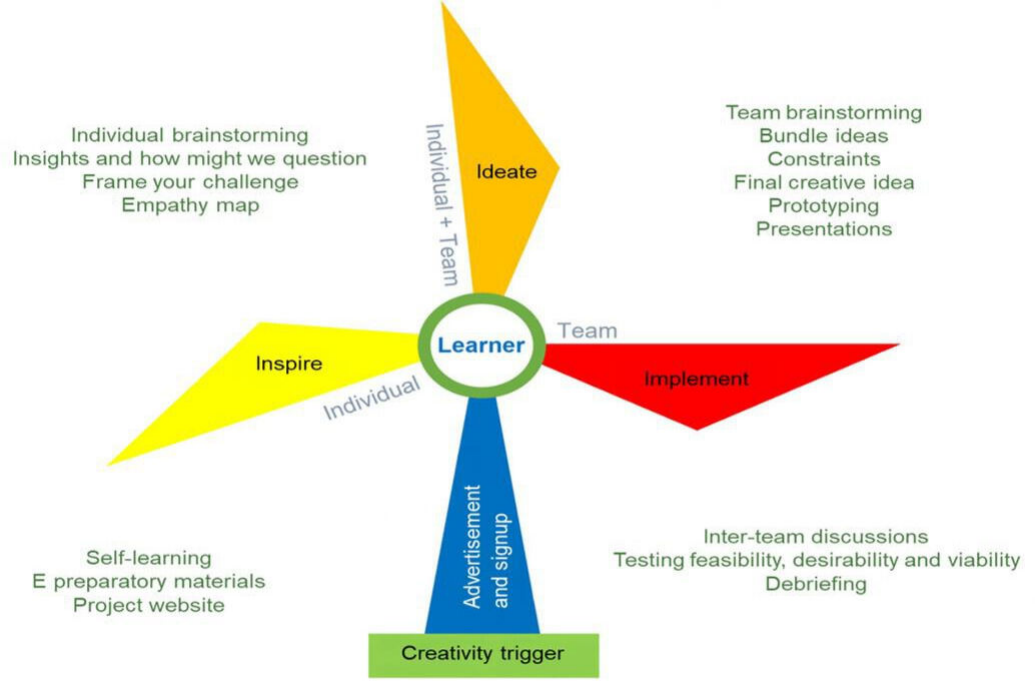
The FLOCC, represented by the 3 phases (Inspire, Ideate, and Implement) of the RDT model. Individual and team activities were done via asynchronous and synchronous learning, respectively. FLOCC: Framework for Learning Online Collaborative Creativity; RDT: rapid design thinking.

The overall flow of FLOCC is captured in Figure 3.

### Ethical Considerations

This study was conducted according to the Declaration of Helsinki, approved by Nanyang Technological University institutional review board (IRB 2020-08-036) before its commencement. Students who participated in this study provided full informed consent. The participants were recruited through class announcements and email correspondence. Participation in the study was voluntary, and it was conducted as an extracurricular activity outside of regular school hours. To show their appreciation for taking part in the study, each participant received SGD $50 (US $38.67). The study data are anonymous, and no images of the manuscript or supplementary material identify individual participants.

### Evaluation of FLOCC

#### Study Participants

We carried out a cross-sectional study to evaluate FLOCC using mixed methods to assess student acceptability of FLOCC. Ayala and Elder [74] define acceptability as “how well an intervention will be received by the target population.” The participants were undergraduate medical students at Lee Kong Chian School of Medicine, Nanyang Technological University Singapore. The TBL is the main pedagogy at Lee Kong Chian School of Medicine, so some TBL elements in FLOCC might feel familiar to the students. We invited the first 3 years of MBBS students for better representation and to reduce the possibility of selection bias. We also asked them to complete the survey fully to ensure that the data is complete and validate the survey results. The evaluation conducted at 2 points with 2 different creativity triggers: study 1 representing the medical (How might we create sustainable health care practices to cater to the growing population?) and study 2 related to engineering, (How might we create sustainable engineering practices to cater to the growing population?).

#### Factors to Measure CC

Mavri et al [75] developed the Assessment Scale for Creative Collaboration (ASCC) to measure social CC in blended learning settings. The ASCC measures three factors: (1) synergistic social collaboration (communication and interaction), (2) distributed creativity (divergent thinking and collective innovation), and (3) time regulation and achievement (time management for shared goals.

We adapted 21 items from the ASCC for this study, to quantify the acceptability of the students toward online CC. We added 3 new items to synergistic social collaboration and 2 new items to time regulation and achievement to better reflect the role of facilitation as a potentially motivating factor toward CC, and the level of engagement of students in both individual and team activities used in the study (refer to Multimedia Appendix 4). Finally, in response to literature which indicates that a learner’s emotional state may also influence CC [76-78], we also created a new fourth factor called self and emotions, which considered 5 items related to confidence, pride, worry and self-consciousness. In summary, there were a total of 31 items across the 4 factors, all in the format of a 7-point Likert scale (1=strongly disagree to 7=strongly agree). In total, 3 free-text questions were also given to collect students’ opinions about the strengths, weaknesses, and suggestions to improve the FLOCC.

#### Delivery

Following informed consent, participants received creativity trigger 1 via email. Students were given 1.5 weeks to complete the individual scaffolds (I1 to I4) before engaging in synchronous online team assignments and discussions held in Microsoft Teams for the team activities of FLOCC. The introduction of the study was conducted by SRM and synchronous part of FLOCC was facilitated by PR who explained the ground rules in the main room (eg, respecting the views of others and do not interrupt), the design thinking processes and how to use the scaffolds for each team activity. Another investigator (SR) was also present to support the digital collaborative platforms (Google Workspace and Microsoft Teams) used in the study.

After this, teams (n≤7 per team) were put into breakout rooms to introduce themselves (ice breaker: 7 min), then brought back to the main room for an explanation of each team events. Teams were given weblinks to Google Sheets to complete T1: bundle ideas in 30 minutes. The toggling between the main to breakout rooms were repeated for the rest of the steps in the following order - T2 and T3: list constraints and final idea (30 min), and T4: prototyping (30 min). A team representative was required to talk about their digital products for 5 min using the prototyping section of the Google Sheets. Then, in the Implementation phase, the final team activity blind testing was carried out (10 min). Here, students were asked to complete T5: blind testing in Google Sheets by answering the free text questions and rating the feasibility, viability, and durability of the other team’s prototype, as explained in the implementation phase. After study 1, participants received a second trigger (sustainability in engineering) for study 2, following similar procedures. A debrief closed each session. The examples of team’s digital products and solutions to triggers 1 and 2 can be found in MultimediaAppendices 5  6, respectively.

#### Data Collection

At the end of each study, the survey (7-point Likert scale items) in Google Forms measured the students’ perceptions of the FLOCC. Students were given 20 minutes to complete the survey.

#### Analysis

Likert-scale questions were analyzed using SPSS (version 28, IBM Corp). Cronbach α was calculated to determine the internal consistency of the survey items within each of the 4 factors (self and emotions, synergistic social collaboration, distributed creativity, and time regulation and achievement). The α value of more than 0.7 was considered satisfactory [78].

After determining the internal consistency of items, the mean and SD, and the percentages of agree, neutral and disagree were consolidated from the 7-point Likert scale at the team, study, and overall cohort levels. This was followed by a Shapiro Wilk and Levene test to determine normality and homogeneity. Data that were normally distributed and equally varied were subjected to an independent samples *t* test and Cohen *d* to determine the statistical significance of means and magnitude (variability) of mean differences [79]. If not normally distributed, data were subjected to the homogeneity of variance for the eligibility of the Mann Whitney U test for statistical calculations based on the mean ranks instead [79]. After that, the eta squared effect size (Ƞ^2^) was calculated to determine the magnitude (variability) of the mean ranks. If eta squared values were found to be negative that is, mean scores of study 2 were larger than study 1, they were reported as absolute values for simplicity purposes. A *P*<.05 was considered statistically significant.

The qualitative free-text comments of study 1 were subjected to thematic analysis to gain insights into the FLOCC. The comments selected from the first study were primarily chosen based on factors such as the presence of significant and representative qualitative data, as well as their suitability for thorough analysis. The analysis was done using Nvivo (version 12, QSR International) via inductive coding by 2 coders (SR from the research team and RC external to the study) [80,81]. Any disagreements were resolved by a third coder (SRM).

## Results

### Demographics

In total, 85 years 1‐3 MBBS students (38 males, 47 females; aged 18‐22 y) participated in 2 studies. A total of 44 students participated in study 1 (21 males, 23 females; aged 18‐22 y), and 41 in study 2 (17 males, 24 females; aged 19‐22). The mean age in each study and the overall sample population (n=85) was 20.3 (SD 0.9) years. All participants completed the survey.

### Internal Consistency of the Questionnaire

Initial Cronbach α for the factors self and emotions, synergistic social collaboration, distributed creativity and time regulation and achievement were 0.42, 0.88, 0.63 and 0.66 respectively. Items 2 “I feel self-conscious when I present my ideas to other people”, 21 “We felt pressured to create something original”, and 27 “We felt pressured to complete the activity on time” were discarded as their contribution lowered the respective factors Cronbach α <0.70 [82]. Upon their removal, the Cronbach α values improved [83], resulting in a refined 28 item instrument (Multimedia Appendix 4) with α values were 0.75 (self and emotions), 0.88 (synergistic social collaboration), 0.75 (distributed creativity) and 0.74 (time regulation and achievement).

### Students’ Perceptions on FLOCC

Overall, the study findings (based on refined 28-item instrument) indicated that most students in the total sample (n=85) had positive views of FLOCC across all 4 factors (70%‐92%) (Table 1). Distributed creativity (mean 5.97, SD 0.14) and synergistic social collaboration (mean 5.68, SD 0.2) displayed higher mean scores, followed by time regulation and achievement (mean 5.44, SD 0.22) and self and emotions (mean 5 SD, 0.3). These trends were consistent in both studies (study 1, n=44; study 2, n=41), where distributed creativity and synergistic social collaboration emerged with higher mean scores than the other 2 factors (Table 2).

**Table 1.** Summary of percentage frequency results segregated by team, study, and overall sample population levels (n=85) for each of the 4 factors (self and emotions, synergistic social collaboration, distributed creativity, and time regulation and achievement).^a^

		Frequency scores (%)					
		Study 1 (n=44)			Study 2 (n=41)		
Factors and levels		Agree	Neutral	Disagree	Agree	Neutral	Disagree
Self and emotions^b^							
	Team 1	66.67	8.33	25	75	10.71	14.29
	Team 2	56.25	12.5	31.25	67.86	14.29	17.86
	Team 3	78.57	10.71	10.71	79.17	0	20.83
	Team 4	67.86	21.43	10.71	70	15	15
	Team 5	54.17	8.33	37.5	65	15	20
	Team 6	68.75	12.5	18.75	80	20	0
	Team 7	46.43	7.14	46.43	83.33	4.17	12.5
	Team 8	79.17	8.33	12.5	—^c^	—	—
	Study overall	64.77	11.36	23.86	74.39	10.98	14.63
Synergistic social collaboration^d^							
	Team 1	91.67	5.56	2.78	94.05	4.76	1.19
	Team 2	97.92	2.08	0	90.48	9.52	0
	Team 3	83.33	4.76	11.9	84.72	9.72	5.56
	Team 4	91.67	8.33	0	90	6.67	3.33
	Team 5	76.39	13.89	9.72	90	6.67	3.33
	Team 6	89.58	8.33	2.08	90	3.33	6.67
	Team 7	84.52	8.33	7.14	77.78	12.5	9.72
	Team 8	94.44	2.78	2.78	—	—	—
	Study overall	87.88	7.01	5.11	88.21	7.72	4.07
Distributed creativity^e^							
	Team 1	94.44	5.56	0	100	0	0
	Team 2	95.83	4.17	0	100	0	0
	Team 3	88.1	9.52	2.38	94.44	5.56	0
	Team 4	92.86	7.14	0	93.33	6.67	0
	Team 5	94.44	2.78	2.78	83.33	10	6.67
	Team 6	95.83	4.17	0	90	10	0
	Team 7	92.86	7.14	0	66.67	25	8.33
	Team 8	100	0	0	—	—	—
	Study overall	93.94	5.3	0.76	90.24	7.72	2.03
Time regulation and achievement^f^							
	Team 1	77.78	11.11	11.11	95.24	2.38	2.38
	Team 2	91.67	4.17	4.17	88.1	7.14	4.76
	Team 3	69.05	16.67	14.29	80.56	11.11	8.33
	Team 4	71.43	21.43	7.14	90	6.67	3.33
	Team 5	61.11	13.89	25	70	26.67	3.33
	Team 6	87.5	0	12.5	83.33	13.33	3.33
	Team 7	64.29	21.43	14.29	86.11	11.11	2.78
	Team 8	88.89	8.33	2.78	—	—	—
	Study overall	74.62	13.64	11.74	85.37	10.57	4.07

a Due to the consolidation of responses into the ”Agree,” ”Neutral,” and ”Disagree” categories for each factor, absolute values could not be provided. The reported percentages are based on these consolidated categories.

b Population overall—Agree: 69.41; Neutral: 11.18; Disagree: 19.41.

c Not applicable.

d Population overall—Agree: 88.04; Neutral: 7.35; Disagree: 4.61.

e Population overall—Agree: 92.16; Neutral: 6.47; Disagree: 1.37.

f Population overall—Agree: 79.80; Neutral: 12.16; Disagree: 8.04.

**Table 2.** Summary of mean results segregated by team, study, and overall sample population levels (n=85) for each of the 4 factors (self and emotions, synergistic social collaboration, distributed creativity, and time regulation and achievement).

Factors and levels		Scores (Likert Scale 1‐7)	
		Study 1 (n=44), mean (SD)	Study 2 (n=41), mean (SD)
Self and emotions^a^			
	Team 1	4.83 (0.63)	5.29 (0.64)
	Team 2	4.81 (0.53)	5.57 (0.53)
	Team 3	5.18 (0.46)	5.33 (0.64)
	Team 4	5.14 (0.18)	4.90 (0.47)
	Team 5	4.38 (0.32)	4.80 (0.58)
	Team 6	4.88 (0.40)	5.65 (0.20)
	Team 7	4.32 (0.12)	5.21 (0.49)
	Team 8	5.17 (0.68)	—^b^
	Study overall	4.84 (0.23)	5.17 (0.40)
Synergistic social collaboration^c^			
	Team 1	5.81 (0.56)	5.82 (0.23)
	Team 2	6.10 (0.33)	5.87 (0.30)
	Team 3	5.43 (0.45)	5.76 (0.26)
	Team 4	5.79 (0.25)	5.67 (0.31)
	Team 5	5.28 (0.38)	5.68 (0.36)
	Team 6	5.44 (0.36)	5.88 (0.51)
	Team 7	5.40 (0.33)	5.39 (0.23)
	Team 8	6.07 (0.37)	—
	Study overall	5.64 (0.23)	5.73 (0.22)
Distributed creativity^d^			
	Team 1	5.94 (0.45)	6.21 (0.16)
	Team 2	6.33 (0.38)	6.38 (0.13)
	Team 3	5.67 (0.17)	6.17 (0.27)
	Team 4	5.93 (0.29)	6.03 (0.42)
	Team 5	5.81 (0.26)	5.70 (0.38)
	Team 6	5.71 (0.32)	6.07 (0.35)
	Team 7	5.93 (0.26)	5.25 (0.29)
	Team 8	6.36 (0.18)	—
	Study overall	5.95 (0.16)	5.99 (0.19)
Time regulation and achievement^e^			
	Team 1	5.17 (0.39)	5.69 (0.25)
	Team 2	5.71 (0.26)	5.69 (0.38)
	Team 3	4.88 (0.31)	5.61 (0.27)
	Team 4	5.24 (0.39)	6.00 (0.27)
	Team 5	4.89 (0.63)	5.37 (0.27)
	Team 6	5.25 (0.32)	5.67 (0.44)
	Team 7	4.93 (0.50)	5.86 (0.33)
	Team 8	5.83 (0.52)	—
	Study overall	5.20 (0.22)	5.70 (0.17)

a Population overall: mean 5 (SD 0.30).

b Not applicable

c Population overall: mean 5.68 (SD 0.20).

d Population overall: mean 5.97 (SD 0.14).

e Population overall: mean 5.44 (SD 0.22).

The Levene’s test showed equality of variances (*P*>.05) for all factors but the Shapiro Wilk test revealed nonnormally distributed data (*P*<.05) for 3 factors (distributed creativity in studies 1 and 2 respectively; and synergistic social collaboration in study 2). This meant that statistical calculations related to these nonnormal distributed groups were subjected to the Mann Whitney U test and eta squared effect size (Ƞ^2^), and the rest of the normally distributed groups were subjected to the independent samples *t* test and Cohen *d* effect size (Table 3). There was no statistical difference on 3 factors (self and emotions, synergistic social collaboration, and distributed creativity) between the study 1 and study 2 (*P*=.12, *P*=.39 and *P*=.35, respectively; Table 3). However, the factor time regulation and achievement was statistically significant (*P*=.001; Table 3). Effect size estimates showed larger variability between studies 1 and 2 in self and emotions and time regulation and achievement (Ƞ^2^=0.34 and 0.72, respectively) than in synergistic social collaboration and distributed creativity (*d*=0.01 for both).

**Table 3.** Summary of statistical results and effect size between studies 1 and 2, for the four individual factors self and emotions, synergistic social collaboration, distributed creativity, and time regulation and achievement.

Factors and study		Sample size, n	Shapiro wilk (*P* value)	Levene test (*P* value)	Independent sample *t* test or Mann Whitney *U* test (*P* value)	Effect size (Cohen *d* or Ƞ^2^)
Self and emotions				.80	.12^a^	−0.34^c^
	Study 1	44	.26			
	Study 2	41	.31			
Synergistic social collaboration				.18	.39^b^	0.01^d^
	Study 1	44	.65			
	Study 2	41	<.01			
Distributed creativity				.48	.35^b^	0.01^d^
	Study 1	44	.04			
	Study 2	41	.01			
Time regulation and achievement				.11	.001^a^	−0.72^c^
	Study 1	44	.44			
	Study 2	41	.90			

a Refer to independent sample *t* test for normally distributed data.

b Refer to Mann Whitney *U* test for nonnormal distributed data.

c Refer to Cohen *d* values based on magnitude of variability of mean values between studies 1 and 2.

d Refer to eta squared (Ƞ2) values based on magnitude of variability of mean ranks between studies 1 and 2.

### Thematic Analysis

Thematic analysis of the free-text responses identified 4 main themes: learning experiences, collaborative responsibilities, perceived skill development, and technical challenges. Quotes are given below to exemplify the open comments.

#### Learning Experiences

Overall, students reported positive experiences on the structure, and opportunities offered by the FLOCC: “Had a good structure that encouraged participation by all team members of the team” (P#54); “Easy to understand…” (P#02); “Good time allocation” (P#29); and “The challenge we had to solve was interesting and relevant…” (P#43).

They also suggested improvements to the FLOCC, indicating their active involvement and participation: “Maybe include other aspects and not just healthcare…” (P#08); “I think that the original question was rather broad…” (P#23); and “Timings given could be a bit longer...” (P#56).

#### Collaborative Responsibilities

Students perceived sharing of thinking processes, information, team diversity and team dynamics as key elements for CC: “We could share our thinking process with each other” (P#01); “It gives students an opportunity to work together and exercise teamwork.” (P#24).

There were mixed opinions related to group formation and group diversity: “Interesting mix of people” (P#25); “Forming groups with students with different backgrounds may allow us to have a more fruitful discussion with different points of view…” (P#02).

#### Perceived Skill Development

Students stated that FLOCC was useful in gaining skills such as design thinking, creativity, communication, and social learning, and self-reflection: “I think that the pre team activity materials were very helpful in helping us understand the design thinking process….” (P#28); “Provides a conducive environment for us to think out of the box and come up with novel ideas for issues we think need to be addressed in the healthcare industry” (P#04); “Team activity allowed me to understand different perspectives I haven’t considered before” (P#35).

#### Technical Challenges

As mentioned earlier, because of the Covid-19 pandemic, FLOCC was carried out online. Feedback indicated students—who were very used to online learning at this time–would have preferred face-to-face interactions: “Feel that the discussions were slightly abrupt as they were carried out online” (P#46); “New faces+online (more difficult to share ideas with)” (P#37); and “A digital medium makes teamwork a little more challenging” (P#58).

## Discussion

### Principal Findings

This paper reported the development and evaluation of a novel FLOCC intervention that draws on TBL, PBL and RDT principles to encourage CC in medical students. This paper provided sufficient details of the intervention to ensure clarity and replicability. The evaluation data from 85 participants indicate a high level of acceptability for FLOCC. Student responses also suggest that the FLOCC offers students with the opportunity to acquire, hone, and demonstrate their CC skills given the right tools, flexibility, and appropriate educational support.

We drew on design thinking principles for the foundation of FLOCC, as recent literature has suggested that design thinking may be an appropriate framework to foster student, patient, and practitioners’ outcomes such as self-efficacy, learning experiences, and academic development [50,52-54]. However, unlike previous studies, we did not base FLOCC exclusively on design thinking principles. Instead, we used a blended approach that draws on sociocultural learning (PBL and TBL) principles to develop a robust conceptual framework. The evaluation results suggest that FLOCC was effective in assisting students in fostering CC skills. Furthermore, the adaptation of the ASCC instrument facilitated the measurement the CC outcomes [75].

Other studies highlight that more supervision may be appreciated when working with students from different educational backgrounds [53], and that facilitators play an important role in creating a positive learning environment [84]. Hence, we incorporated a specific survey item to measure students’ perceptions about the quality of the facilitation received from instructors leading the FLOCC. The qualitative data from the open comments indicated that students valued effective facilitation. Similarly, we added a factor “Self and Emotions.” Although feelings and emotions can influence the quality of creative outputs [39,47], these are rarely assesses in design thinking models and CC. The importance of these factors to learning was reflected in the students’ open comments.

To the authors’ knowledge, this is the first study to measure the 4 factors of CC in the context of medical education. This study’s new observations were that students (overall population) perceived higher emphasis on Distributed Creativity (78/85, 92%) and Synergistic Social Collaboration (75/85, 88%) followed by time regulation and achievement (68/85, 80%), and self and emotions (59/85, 70%). This suggests that PBL, TBL, and RDT theoretical perspectives, along with selected technologies of FLOCC, enabled a virtual environment where participants could actively engage in teams to practice collaboration, communication, collective knowledge generation, design thinking, and collaborative problem solving. These observations may come from the ability to relate their educational background and experiences to the creativity trigger, and the perceived proximity from working virtually with other students in the same discipline [85]. On the other hand, previous studies have reported forming interdisciplinary and multidisciplinary teams can be beneficial, depending on the nature of the task [86,87]. Therefore, it would be interesting to provide a trigger that could be beyond students’ educational background and form multidisciplinary teams to measure the effects of the FLOCC process on outcomes of CC.

When comparing 2 studies, results showed no significant differences in any of the variables tested, except hose pertaining to time regulation and achievement. This suggests that FLOCC is successful in fostering teamwork and scaffolding the multifaceted creative process, regardless of problem type, time periods, or study population. Based on these findings, the FLOCC structure is robust and may be safe for use in other contexts. Regarding differences in perceived time regulation and achievement, we believe that these differences are a result of the technical difficulties encountered in study 1 and the fact that certain students may find the online environment challenging. Previous research indicated that challenges associated with technology, students, and facilitators are not necessarily barrier to CC, but rather as opportunities to enhance it through problem solving, being considerate of others, and appreciating the teacher’s effort [30].

### Limitations and Future Research Recommendation

There were several limitations in this study. First, it involved volunteer medical students from a single medical school in Singapore. The views of more students at different levels of medical education, and from other medical schools could be gathered in future research. Second, participants were experienced with TBL, self-selecting and may have had more of an interest in design thinking and collective collaboration than typical medical students. Consequently, participants of this study may have different views on group work and differing group decision-making skills. However, the sample size was reasonable for an exploratory “proof of concept” and acceptability study. As is often the case with online education, it is difficult to separate out student satisfaction with the content and pedagogy of teaching as opposed to the quality of internet connections and technical difficulties. The current evidence is mainly based on the students’ self-reported data about their experience with FLOCC. Future research could investigate whether FLOCC is effective and efficient in nurturing CC skills for medical education or practice, as compared to established pedagogies such as PBL and TBL.

Realistically, CC skills cannot be developed in a short time. Therefore, we suggest that the principles underpinning FLOCC should ultimately be used in a curricular (rather than one-off) basis if fostering collective creativity is a priority in medical education.

### Implications

FLOCC in the digital environment opened up new possibilities such as the opportunity to understand the “precise mechanisms” needed to acquire and apply new or innovative knowledge. The framework also underpinned the monitoring of individual and team creativity processes in an organized manner. This is important because if the collection of data is unorganized, comprehending and analyzing the stakeholders’ reflections would become challenging and almost impossible to measure and to provide feedback [56]. In the context of medical education and practice, this might influence the past, present and future interpretations of appropriate patient care [64]. Educators can leverage FLOCC as a model to design and modify learning activities that further CC and connect the classroom to real-world settings. Testing this framework face-to-face, with other educational intra and interdisciplines (engineering, sciences, business, and others), and a larger sample size may also form part of future studies.

### Conclusions

The FLOCC model, which integrates the design thinking and sociocultural learning methods (PBL and TBL), holds considerable promise as a framework for cultivating CC in medical education. Further research is needed to evaluate its effectiveness across intermultidisciplinary teams and diverse creativity triggers, thereby uncovering its full potential.

## Supplementary material

10.2196/50912 Multimedia Appendix 1 Hybrid characteristics of the Framework for Learning Online Collaborative Creativity (FLOCC) based on the strengths of team- and problem-based learning (TBL and PBL), and rapid design thinking (RDT).

10.2196/50912 Multimedia Appendix 2 Conceptual underpinnings of the Framework for Learning Online Collaborative Creativity (FLOCC).

10.2196/50912 Multimedia Appendix 3 Pictorial illustration of website.

10.2196/50912 Multimedia Appendix 4 The FLOCC (Framework for Learning Online Collaborative Creativity), represented by the 3 phases (Inspire, Ideate and Implement) of the rapid design thinking (RDT) model. Individual and team activities were done via asynchronous and synchronous learning, respectively.

10.2196/50912 Multimedia Appendix 5 Examples of team’s digital products and solutions to trigger 1.

10.2196/50912 Multimedia Appendix 6 Examples of team’s digital products and solutions to trigger 2.

## Abbreviations

ASCC: Assessment Scale for Creative Collaboration
CC: collaborative creativity
FLOCC: Framework for Learning Online Collaborative Creativity
PBL: problem-based learning
RDT: rapid design thinking
TBL: team-based learning
