# Supplementary material for: Developing and Testing a Framework for Learning Online Collaborative Creativity in Medical Education: Cross-Sectional Study
Source: JMIR Form Res. 2025 Jun 5;9:e50912. doi: 10.2196/50912 (PMC12161162; doi:10.2196/50912)
Supplement: Multimedia Appendix 1 [file formative-v9-e50912-s001.pdf]

Supplementary Material 2. Contents of individual Excel Spreadsheet.

GENERAL DATA	
**What is your challenge/question?**	
**Designed for (Who is the stakeholder involved? Name, age, gender, job, etc):**	
**Designed by:**	
**Date:**	

EMPATHY MAP					
**What does he/she think and feel?**	**What does he/she hear?**	**What does he/she see?**	**What does he/she say and do?**	**Pain**	**Gain**
What really matters, Major preoccupations, Worries and aspirations, etc.	What friends say, what boss says, what influencers say, etc.	Environment, friends, social medial, websites, magazines, what the market offers, etc.	Attitude in public, appearance, behaviour towards others, etc.	Fear, frustrations, obstacles	"Wants"/needs, measures of success, Goals

Frame your challenge

What is the problem you are trying to solve?	Enter Text
Try to frame the problem as a question.	Enter Text
What is the impact you are trying to have?	Enter Text

What are some possible solutions?	Enter Text
Write some of the context and constraints you are facing?	Enter Text
Does your original question need a tweak? Try again.	Enter Text

Turning Insights into How Might We Questions

Example
Insight: Students want more cost-effective options when commuting.
How might we help students save transport costs when they commute to the university?

Insight 1:
How might we (enter text)

Insight 2:
How might we (enter text)

Insight 3:
How might we (enter text)

You may add more..

Brainstorming

Example

How might we help students save transport costs when they commute to the university?

Idea:
1. Student seasonal transport pass
2. Provide affordable accommodation
3. Provide university buses that runs in popular residential suburbs/from train stations to the university
4. Car pooling

What is the 'How might we' question?	How might we (enter text)
What is the idea?	1
	2
	3
	4

You can add more..
