# Supplementary material for: Developing and Testing a Framework for Learning Online Collaborative Creativity in Medical Education: Cross-Sectional Study
Source: JMIR Form Res. 2025 Jun 5;9:e50912. doi: 10.2196/50912 (PMC12161162; doi:10.2196/50912)
Supplement: Multimedia Appendix 2 [file formative-v9-e50912-s002.pdf]

Supplementary Material 1. Pictorial illustration of website.

Reimagine Medicine and Engineering:

Stimulating collaborative creativity using Team-Based Approach

MOE-TRF Project | NTU IRB Number: IRB-2020-08-036

This is an NTU-secured Google Site. For access, please sign out from all personal google accounts (e.g. Google Drive, Gmail) and login again with NTU credentials.

1 Register as a Participant

Registration form

2 Consent

Consent form

3 Introductory Session to Design Thinking

Watch the Pre-recorded video (mp4 format)

4 Individual Thoughts

Step 1: DOWNLOAD the 'Individual Thoughts' excel sheet

Step 2: SUBMIT the completed 'Individual Thoughts' excel sheet

5 Team Discussions

To be announced via E-mail.

6 Other Enquiries

Troubleshoot Google Workspace accounts using NTU credentials

Please email Asst. Prof. Sreenivasulu Reddy Mogali (sreenivasulu.reddy@ntu.edu.sg) or Dr. Shairah Radzi (shairah.radzi@ntu.edu.sg) should you have any other questions regarding the study.

Nanyang Technological University Singapore 2021. All Rights Reserved.
