# Supplementary material for: Developing and Testing a Framework for Learning Online Collaborative Creativity in Medical Education: Cross-Sectional Study
Source: JMIR Form Res. 2025 Jun 5;9:e50912. doi: 10.2196/50912 (PMC12161162; doi:10.2196/50912)
Supplement: Multimedia Appendix 3 [file formative-v9-e50912-s003.pdf]

Supplementary Material 4. Contents of team Google Sheets.

Bundle Ideas

List all the ideas from all team members during individual brainstorming.

You may come up with new ideas as a team through this process.

You may group ideas together to form a concept or a theme.

You may remove duplicated ideas.

List Constraints

What are the constraints or barriers that your team identified to select a final idea? This is open to your team's interpretation!

Eg. Ability of team members, technology, etc. Think of any other constraints and document it here!

Final Idea

What is the criteria your team used to choose a final idea?
How did your team select the final idea?
Eg. Must haves/wants vs. needs etc.
How would you rate the final idea as a team on a scale of 1 to 10? (1 = Least Satisfied to 10 = Most Satisfied)

Explain why your team feels this way.

Write your final idea.

Prototyping

(If you have drawings/images/storyboards/sketches or any other types of presentation, please upload them into this sheet. Open to the intepretation of the team!)

Questions to ponder: How would you describe your prototype? What are the features you would like to have in the prototype? Who is your end-user?

Blind Testing: How does your team know that the prototype will address the assigned question?

What do you like about the prototype?
What do you dislike about the prototype?

What suggestions can you give to improve the prototype?
Desirability (will people like it?) On a scale of 1 to 10, please rate the prototype. (1 = least desirable to 10 = most desirable)

Feasibility (Can we make it?) On a scale of 1 to 10, please rate the prototype. (1 = least feasible to 10 = most feasible)

Viability (Can it be viable?) On a scale of 1 to 10, please rate the prototype. (1 = least viable to 10 = most viable)
