# Supplementary material for: Developing and Testing a Framework for Learning Online Collaborative Creativity in Medical Education: Cross-Sectional Study
Source: JMIR Form Res. 2025 Jun 5;9:e50912. doi: 10.2196/50912 (PMC12161162; doi:10.2196/50912)
Supplement: Multimedia Appendix 4 [file formative-v9-e50912-s004.docx]

**Supplementary Material 3.**

Table 1. Survey items used in the study. Items in black text adapted from Mavri et al., (2020). Items in blue text were newly developed by the research team. The ‘Agree’ was consolidated from the Likert Scale of 5 to 7 (5 = somewhat agree to 7 = strongly agree). The ‘Disagree’ was consolidated from the Likert Scale of 1 to 3 (1 = strongly disagree to 3 = somewhat disagree). Items marked in asterisk (*) were removed from the instrument due to Cronbach alpha values below 0.7.

| **Survey Items** |
| --- |
| **Self and Emotions** |
| 1. I feel confident to come up with novel ideas. |
| 2. I feel self-conscious when I present my ideas to other people. * |
| 3. I am not worried if I fail in being creative. |
| 4. It was easy for me to brainstorm on ideas. |
| 5. I take pride in being a creative person. |
| **Synergistic Social Collaboration** |
| 6. It was fun to work as a team. |
| 7. I had a good idea of what the others in the team knew that is relevant to the activity. |
| 8. We were able to discuss questionable/controversial ideas to each other. |
| 9. We were able to share and discuss our early ideas with each other. |
| 10. Our team had the necessary knowledge to be able to complete the activity. |
| 11. We were interested in the team activity. |
| 12. We felt very absorbed in the team activity. |
| 13. We trusted each other. |
| 14. Everyone wanted to make a successful prototype. |
| 15. We felt a sense of belonging together. |
| 16. We understood each other's viewpoints. |
| 17. The facilitator played an important role in motivating us to be engaged in the activity. |
| **Distributed Creativity** |
| 18. We were able to generate novel ideas in response to the activity. |
| 19. Although we can disagree on certain things, we managed to understand each other's perspective. |
| 20. We weren't always certain about how to carry out the activity which led us to explore different possibilities. |
| 21. We were able to communicate with each other. |
| 22. We felt pressured to create something original. * |
| 23. We were able to share information with other team members digitally (shared documents etc). |
| 24. We could see or find out what other people know or were thinking about. For example, we could draw, write or build things on the computer that other team members could see and/or read. |
| **Time Regulation and Achievement** |
| 25. I organized my time for learning well. |
| 26. The length of the team activities was adequate. |
| 27. The team activity enabled us to express our emotions. |
| 28. We felt pressured to complete the activity on time. * |
| 29. We went beyond the set activity. |
| 30. Our team organized our time for learning well. |
| 31. Between us we used a lot of imagination. |
